# Contrasting Physiological Responses of Two Populations of the Razor Clam *Tagelus dombeii* with Different Histories of Exposure to Paralytic Shellfish Poisoning (PSP)

**DOI:** 10.1371/journal.pone.0105794

**Published:** 2014-08-25

**Authors:** Jorge M. Navarro, Katerina González, Barbara Cisternas, Jorge A. López, Oscar R. Chaparro, Cristian J. Segura, Marco Córdova, Benjamín Suárez-Isla, María J. Fernandez-Reiriz, Uxio Labarta

**Affiliations:** 1 Instituto de Ciencias Marinas y Limnológicas, Universidad Austral de Chile, Valdivia, Chile; 2 Escuela de Acuicultura, Universidad Católica de Temuco, Temuco, Chile; 3 Laboratorio de Toxinas Marinas, Facultad de Medicina, Universidad de Chile, Santiago, Chile; 4 Instituto de Investigaciones Marinas, Consejo Superior de Investigaciones Científicas, Vigo, España; University of Connecticut, United States of America

## Abstract

This study describes the physiological performance of two populations of the razor clam *Tagelus dombeii* from two geographic areas with different histories of exposure to paralytic shellfish poisoning (PSP) linked to the toxic dinoflagellate *Alexandrium catenella*. Clams from Melinka-Aysén, which are frequently exposed to PSP, were not affected by the presence of toxins in the diet. However, clams from Corral-Valdivia, which have never been exposed to PSP, exhibited significantly reduced filtration activity and absorption, affecting the energy allocated to scope for growth (SFG). Ammonia excretion and oxygen uptake were not affected significantly by the presence of *A. catenella* in the diet. Measurements of energy acquisition and expenditure were performed during a 12-day intoxication period. According to three-way repeated measure ANOVAs, the origin of the clams had a highly significant effect on all physiological variables, and the interaction between diet and origin was significant for the clearance and absorption rates and for the scope for growth. The scope for growth index showed similar positive values for both the toxic and non-toxic individuals from the Melinka-Aysén population. However, it was significantly reduced in individuals from Corral-Valdivia when exposed to the diet containing *A. catenella*. The absence of differences between the physiological response of the toxic and non-toxic clams from Melinka-Aysén may be related to the frequent presence of *A. catenella* in the environment, indicating that this bivalve does not suffer negative consequences from PSP. By contrast, *A. catenella* has a negative effect on the physiological performance, primarily on the energy gained from the environment, on *T. dombeii* from Corral-Valdivia. This study supports the hypothesis that the history of PSP exposure plays an important role in the physiological performance and fitness of filter feeding bivalves.

## Introduction

Harmful algae blooms (HABs) are cosmopolitan phenomena that cause serious public health problems. HABs are also detrimental to aquatic organisms, with negative effects on their physiological functions and also on aquaculture activities. During recent decades, HABs producing paralytic shellfish poisoning (PSP) have increased worldwide [1], [2], and dinoflagellates of the genus *Alexandrium* are the primary producer of the paralytic toxin. This toxin may accumulate in different taxa of the marine food chain, including bivalves, zooplankton, crustaceans, and gastropods [3]. Several physiological and behavioral effects have been described in marine copepods and bivalves exposed to diets containing PSP, such as reductions in ingestion, metabolism and growth rates [4], [5], [6], [7], [8] and changes in the burial patterns of infaunal bivalves [9]. However, the responses to PSP may be influenced by the history of exposure to the toxin [9]. The evolution of grazer adaptation to toxic algae, in both the ocean and freshwater, has been well established [8]. Populations of the copepod *Acartia hudsonica* historically exposed to PSP produced by bloom of dinoflagellates of the genus *Alexandrium spp*., exhibit enhanced feeding and growth rate, as well as fecundity [10], [7], compared to populations never exposed to PSP. Hairston et al. [11] showed that the freshwater grazing cladoceran *Daphnia galeata* evolved a selection response to increased abundance of toxic cyanobacteria in its environment. *Mya arenaria* clams from areas frequently exposed to toxic dinoflagellate blooms are less affected by PSP than specimens from areas that have not been previously exposed to PSP [9]. According these authors, the different responses of bivalves to toxins are related to nerve sensitivity, where resistance to the toxin is caused by a mutation of an amino acid that causes a decrease in the affinity of saxitoxin at the sodium channel pore of the cell membrane. Thus, the presence of PSP in the environment can act as an agent of natural selection, leading to increased resistance of the bivalves to the toxin, with a smaller impact on behavioral and physiological responses. This response favors an increased concentration of toxin in the bivalve, thereby increasing the risk to humans. The expansion of toxic algal blooms to geographical areas not previously affected may result in structural changes in the communities and ecosystem because toxins produced by dinoflagellates can cause significant mortalities in bivalve populations with no history of exposure to PSP [12], [13]. It is possible to find individuals with different physiological and behavioral responses depending on the history of exposure to toxic events [14], [9]. A study that analyzed the digestive enzymatic activity and absorption efficiency in the razor clam *Tagelus dombeii* upon exposure to *Alexandrium catenella* [15] showed that a feeding history of exposure to *A. catenella* was reflected in the digestive responses of *T. dombeii*.

In southern Chile, the dinoflagellate *Alexandrium catenella* has expanded its geographical distribution during the last several decades, with frequent blooms in the Aysén and Magallanes regions and extending north to the center of the Chiloé Island [16], [17], [18]. This geographical region has numerous species of commercially important bivalves, where the extraction and consumption of bivalves have been significantly reduced by the temporary or indefinite closure of areas where bivalves remain toxic with PSP throughout the year. We used the bivalve *Tagelus dombeii*, an infaunal species with a broad latitudinal distribution and that inhabits soft sediments of the tidal and subtidal zones of south Chile, as a model. Razor clam fishery represents greater than 5% of all commercially important benthic resources of Chile. Navarro et al. [19] studied the feeding behavior of *T. dombeii* and concluded that this bivalve behaves as a suspension-feeder when immersed, which indicates that algal blooms are part of its diet. Because of the wide geographical distribution of this species along the Chilean coast, there are populations in southern Chile exposed frequently to PSP, unlike the majority of other populations located in the north, which do not have a history of PSP exposure.

The present study looks at how historical exposure to a toxic dinoflagellate may affect physiological performance and fitness of specimens of *Tagelus dombeii* from two populations from different geographic areas.

## Materials and Methods

### Animal Collection and diet preparation

Adult specimens of *Tagelus dombeii* were collected from the natural banks at Corral-Valdivia (39° 53'S, 73° 25'W; no previous PSP exposition) and Melinka-Aysén (43° 52'S, 73° 45'W; previous PSP exposition). No specific permissions were required to collect the experimental clams from Corral-Valdivia. However, a special permit from the Regional Health Department was required to collect clams from Melinka-Aysén. Individuals ranging from 50 to 60 mm (mean 53.7±4.5 mm) shell length were maintained for one week before the measurements were initiated in aquaria at 14°C, 30 psu. The clams were buried in fine sediment collected from the same location where specimens were collected and fed continuously with a diet containing (by weight) 60% of the microalga *Isochrysis galbana* and 40% inorganic sediment (1.5 mg L^−1^). The monoclonal non-axenic *Alexandrium catenella* (strain ACC02; 32–36 µm spherical diameter) used for the experiments was isolated from the Aysén Region of Chile and was cultivated in 0.45 µm filtered seawater enriched with “L1” algae culture medium [20]. The toxicity of *A. catenella* cells was quantified using the electro-physiological test of Vélez et al. [21], and a mean value from 15 samples was obtained. The microalgae *Isochrysis galbana* was cultivated using f/2 medium [22]. Both species of algae were harvested during the exponential growth phase. Sediment was added to the diets to emulate the organic/inorganic fractions of the natural suspended particulate matter recorded in the field [23]. This sediment was collected from the upper centimeter of the Yaldad tidal flat in south Chile, passed through a 40-µm mesh sieve, rinsed with distilled water, and ashed in a muffle furnace at 450°C for 12 h to eliminate the organic fraction. After ashing, the sediment was resieved (40-µm sieve) to eliminate the sediment aggregates.

### Experimental Design

Three replicates of 25 individuals each were maintained in 8 L aquaria. The clams were permanently buried in the sediment collected from their natural habitat and fed with toxic diet (by weight: 50% *Alexandrium catenella*, 10% *Isochrysis galbana* and 40% inorganic sediment) for a period of 12 days. In parallel, three other similar aquaria were maintained as controls, with the same number of individuals in each group (n = 25) that were fed the non-toxic diet (60% *I. galbana* and 40% inorganic sediment). The diets were continuously supplied with a Masterflex L/S peristaltic pump. The quantity of food provided daily was equivalent to 2% (ca. 14 mg/day/clam) of the dry weight of the soft tissue of the experimental animals. For each sampling date, all physiological processes were measured on the same clam (one from each aquarium, 3 toxic and 3 non-toxic), beginning with clearance rate; feces produced during that time were used to measure the absorption rate. Once the clearance rate experiments were completed, ammonia excretion and oxygen uptake were determined. All following sections are based on this experimental design. Once all measurements were done, the clams were sacrificed to determine the soft tissue weight. To estimate the total weight and organic content of the diets, a known volume of each was filtered, in triplicate, through Whatman 47-mm-diameter glass fiber GF/C filters, which were previously washed, burnt and weighed. A blank filter and those containing the samples were washed with an isotonic solution of ammonium formate to remove the salt and prevent cell lysis. The filters were dried at 100°C for 24 h, weighed, burnt at 450°C for 3 h and reweighed after cooling in a desiccator.

### Physiological measurements

The feeding, absorption, excretion and respiration rates were monitored throughout the experiment on days 0, 1, 2, 3, 5, 8 and 12 in different clams exposed to both the toxic and non-toxic diets. All experiments were performed under controlled temperature (14°C) and salinity (30 psu) conditions.

### Clearance rate (CR)

The CR was estimated in a static system homogenized by aeration and using a food concentration ca. 2.0 mg L^−1^ dry weight (Table 1). Each experimental aquarium (1.0 L volume) contained a single clam, and the reduction in particle concentration in the aquaria was monitored periodically with a Beckman model Z2 particle counter equipped with a 100 µm aperture counting tube. The decrease in particle concentration in the experimental aquaria was maintained between 10 and 40% in relation to the initial concentration and was measured every 30 min for 4 h, with replacement of the consumed food. To test for any growth or cell sedimentation during the feeding measurements, a control aquarium without clams was maintained. The CR (l h^−1^ ind^−1^) was calculated following the method of Coughlan [24].

**Table 1:** Characterization of toxic and non-toxic diets supplied to the razor clam *Tagelus dombeii*.

Diet	Total dry weight	Organic dry weight		Sediment	*I.galbana*	*I.galbana*	*A.catenella*	*A.catenella*	Toxicity
	(mg l^−1^)	(mg l^−1^)	%	(mg l^−1^)	(cells l^−1^)	(mg l^−1^)	(cells l^−1^)	(mg l^−1^)	(pmol STX Eq l^−1^)
Toxic (50% *A.catenella*)	1.99±0.06	1.21±0.05	60.80±0.95	0.80±0.02	6.64×10^6^	0.20±0.01	1.98×10^5^	0.99±0.03	2039
Non-toxic (60% *I.galbana*)	1.95±0.19	1.08±0.09	56.08±1.24	0.78±0.08	39.1×10^6^	1.17±0.12	0	0	0

### Absorption rate (AR)

The AR was calculated as the product of the absorption efficiency and the organic ingestion rate. The absorption efficiency data were obtained from Fernández-Reiriz et al. [15], who performed a parallel study using the same experimental specimens on the digestive enzyme activity of *Tagelus dombeii*.

### Ammonia excretion (VNH_4_-N)

The clams fed toxic and non-toxic diets were placed individually in glass beakers containing filtered (0.45 µm) seawater. One additional beaker containing filtered seawater but no clams was used as a control. All beakers were maintained at the experimental temperature by submersion in a thermostatic water bath. After 2 h, water samples from each beaker were removed and analyzed for ammonia–nitrogen according to Solórzano [25].

### Oxygen uptake (VO_2_)

Oxygen uptake was determined individually in 1.0 L chambers sealed for 60 min. Measurements of the oxygen dissolved in the sea water were recorded after this period of time to prevent the oxygen concentration from falling below 70% saturation. A chamber of similar volume without bivalves was used as the control. The initial and final concentrations of oxygen were measured on 50 ml samples using the micro-Winkler method.

### Scope for growth (SFG)

The measurements of the energy available for growth and reproduction (SFG) were calculated using the equation given by Widdows [26] after converting all physiological rates to energy equivalents (J h^−1^):
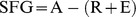
Where A = energy absorbed: 1 mg organic matter of food = 21 J [27]; R = oxygen uptake: 1 ml O_2_ = 19.9 J [28] and E = ammonia excretion: 1 µg NH_4_–N = 0.0249 J [28].

### Statistical analysis

The diets and individual physiological rates were compared by a one-way analysis of variance (ANOVA). The different physiological processes were measured in each tank over time; therefore, it was necessary to apply an analysis of variance for repeated measures [29], which considers the temporal dependency between samples from the same aquarium. Three-way repeated measure ANOVAs (tank as random factor) were performed to analyze the effects of diet (toxic and non-toxic), origin (Corral-Valdivia and Melinka-Aysén), and time of exposure (TE) on the clearance, absorption, ammonia excretion and respiration rates, and scope for growth. When the interaction was significant and involved the time of exposure (TE), a two-way ANOVA for repeated measures was used. The normality and homoscedasticity of the data were tested using the Kolmogorov-Smirnov and Bartlett tests, respectively [30]. The statistical analyses were performed using the R 3.0.2 software (R Development Core Team 2011).

### Animal Research

This study was performed in the Laboratory of Marine Ecophysiology of the Universidad Austral de Chile and the species involved in this research is not endangered or protected. The protocol was approved by the Committee on the Bioethics of Animal Research of the Universidad Austral de Chile (Permit Number: 26-2011).

## Results

### Experimental diets

The characteristics of the toxic and non-toxic diets are summarized in Table 1. No significant differences (P>0.05) were observed between the total weight of the toxic diet (1.99±0.06 mg l^−1^) and the non-toxic diet (1.95±0.19 mg l-1), nor among their organic fractions (toxic: 60.80% and non-toxic: 56.08%). The mean concentration of toxin in *A. catenella* (strain ACC02) was 10.3±0.91 fmol STX eq/cell. The concentration of *A. catenella* cells in the experimental diet was 1.98×10^5^ cells L^−1^, resulting in a concentration of saxitoxin equivalent to 2039 pmol L^−1^ (Table 1).

### Physiological responses

**Figure 1:**
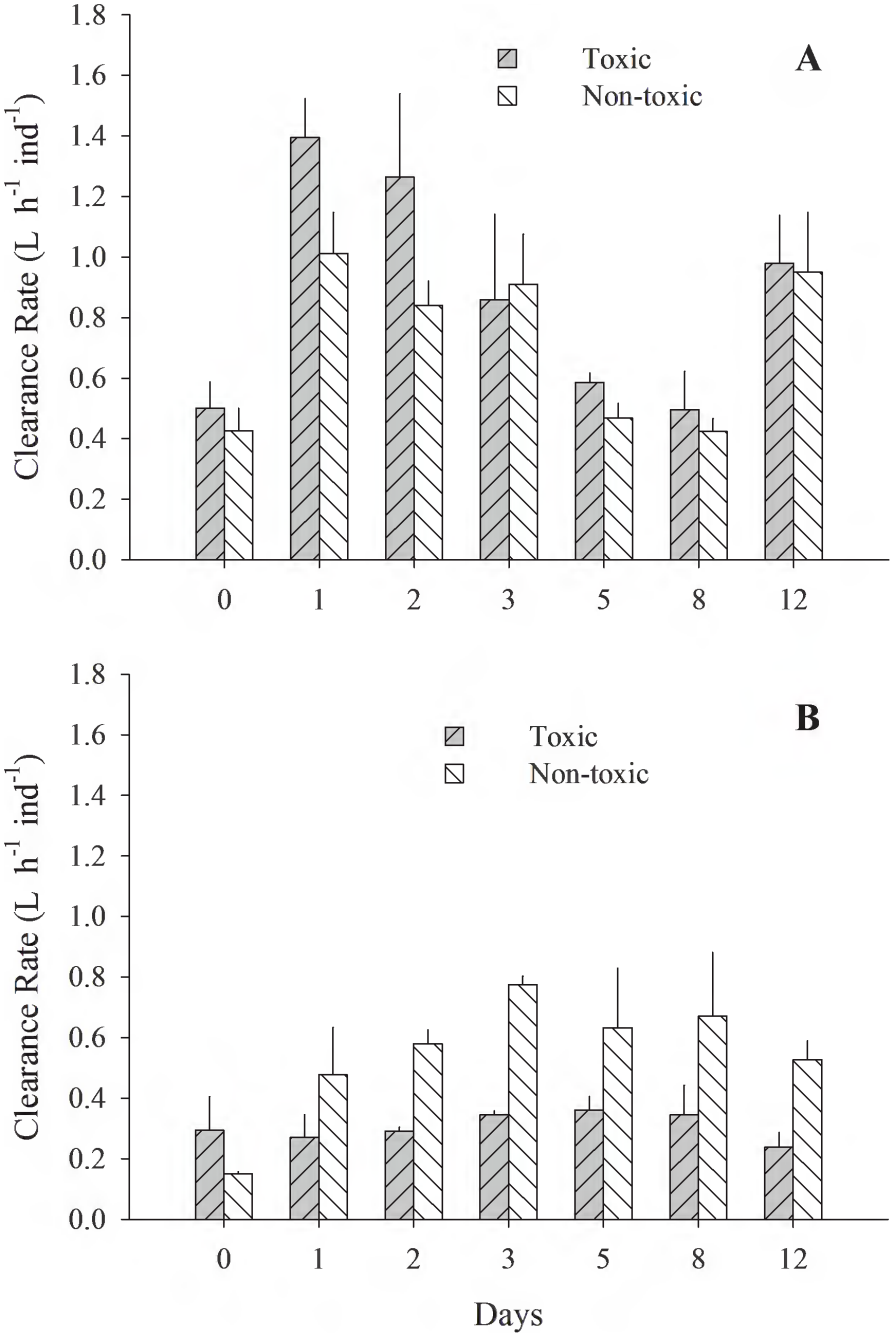
*Tagelus dombeii*. Clearance rate measured for a period of 12 days in individuals with different histories of exposure to PSP and exposed to toxic and non-toxic diets (3 replicates per experimental group at each sampling time). A, Melinka, Aysén (with previous PSP exposure); B, Corral, Valdivia (without previous PSP exposure).

**Figure 2:**
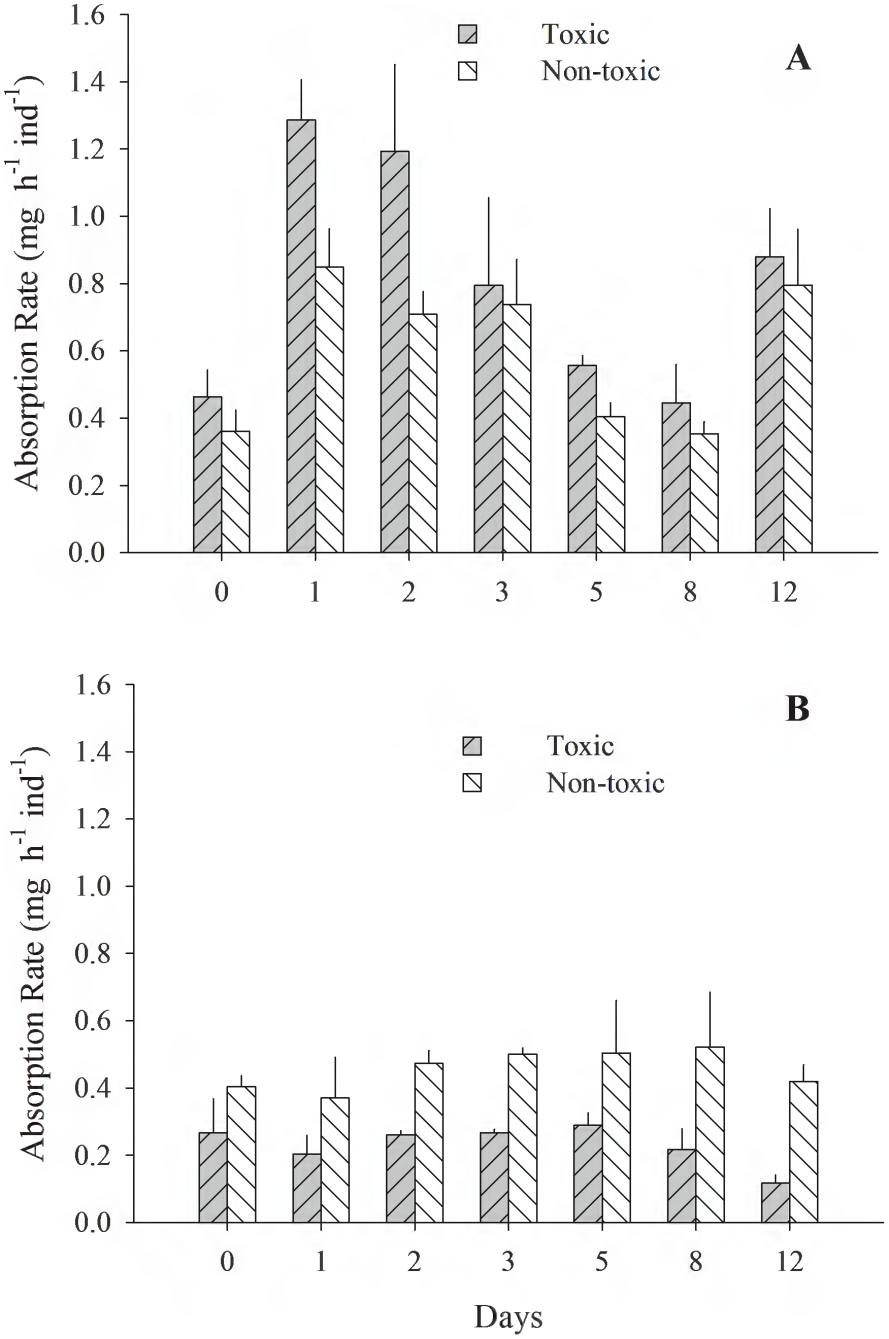
*Tagelus dombeii*. Absorption rate measured for a period of 12 days in individuals with different histories of exposure to PSP and exposed to toxic and non-toxic diets (3 replicates per experimental group at each sampling time). A, Melinka, Aysén (with previous PSP exposure); B, Corral, Valdivia (without previous PSP exposure).

**Figure 3:**
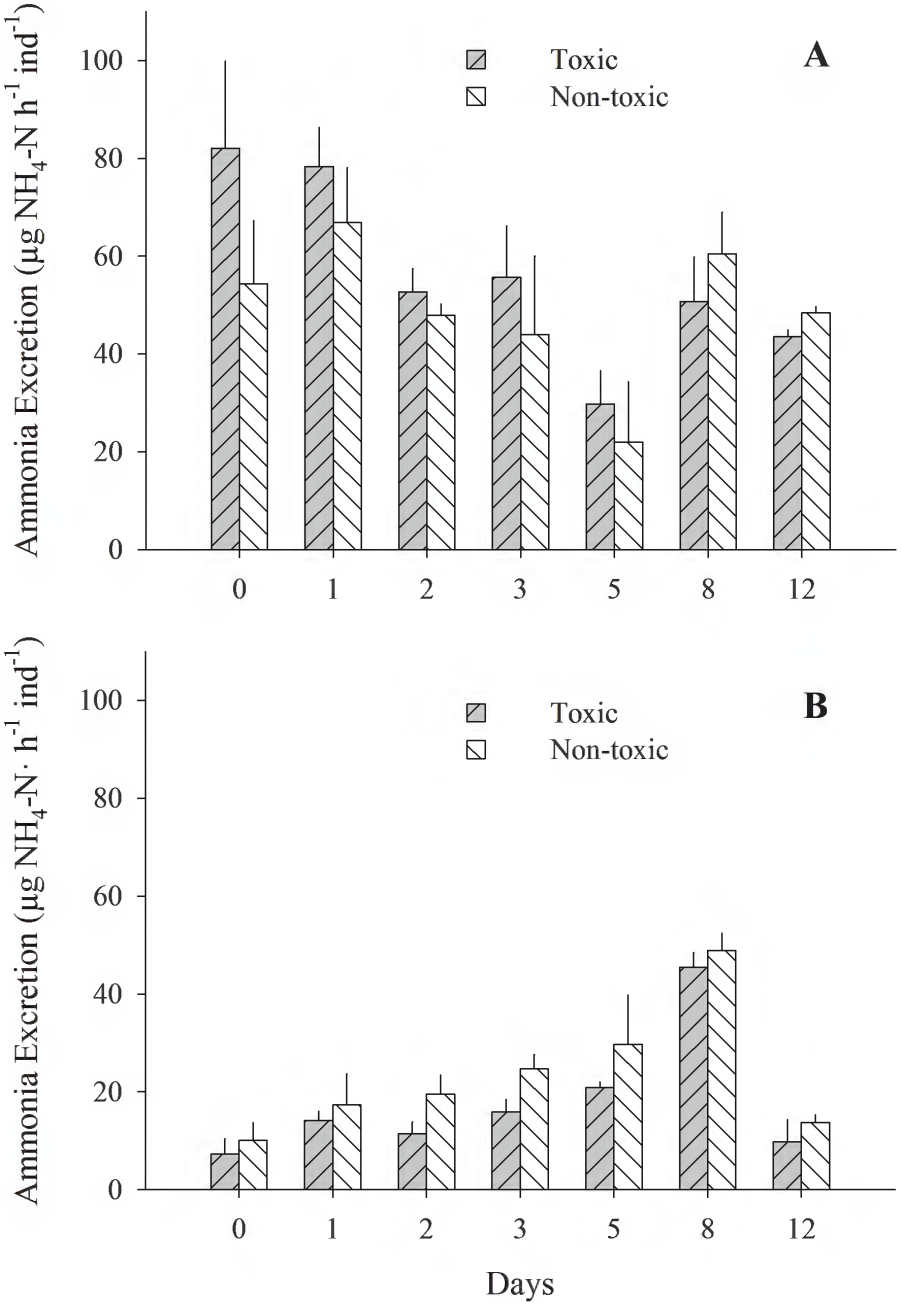
*Tagelus dombeii*. Ammonia excretion measured for a period of 12 days in individuals with different histories of exposure to PSP and exposed to toxic and non-toxic diets (3 replicates per experimental group at each sampling time). A, Melinka, Aysén (with previous PSP exposure); B, Corral, Valdivia (without previous PSP exposure).

**Figure 4:**
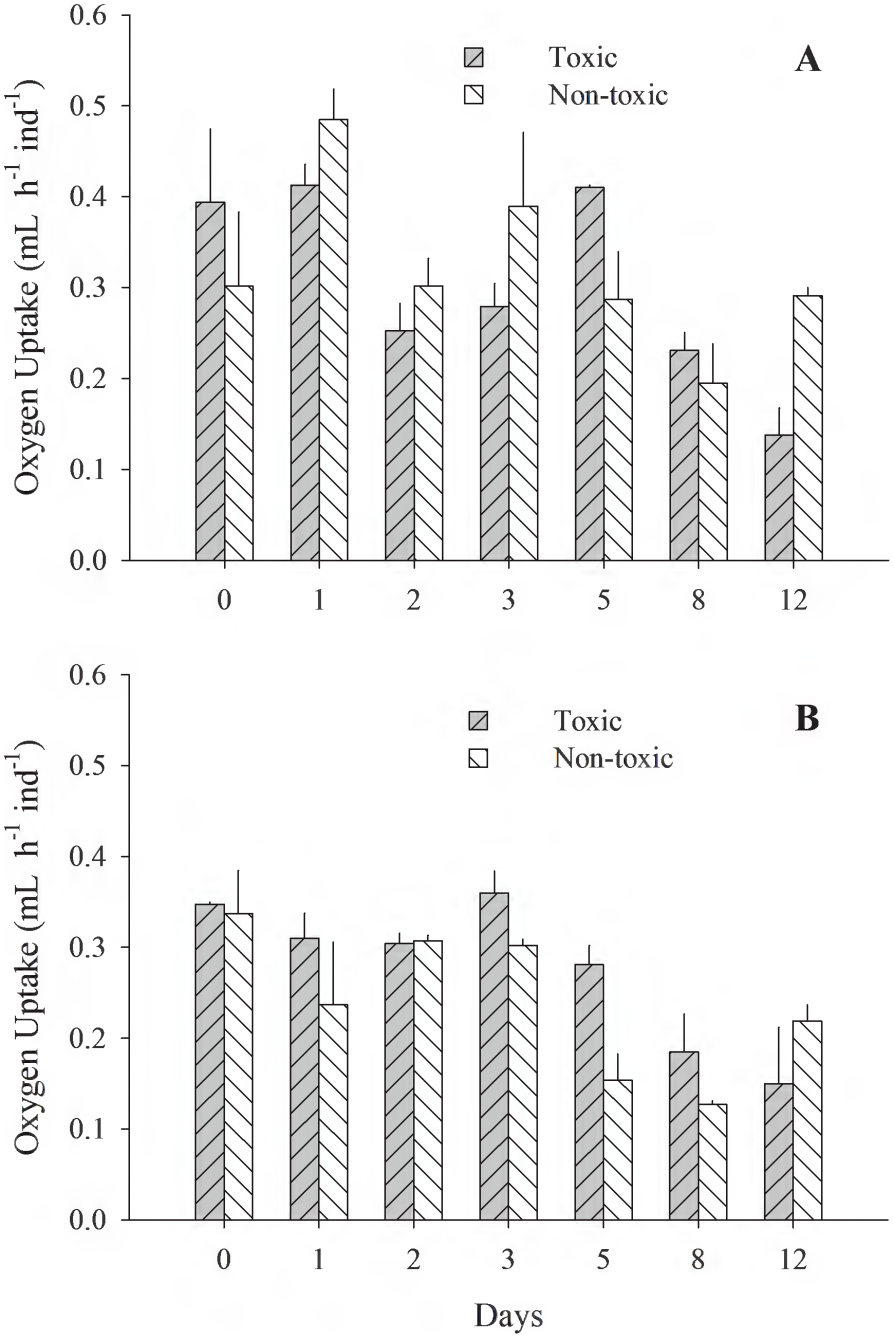
*Tagelus dombeii*. Oxygen uptake measured for a period of 12 days in individuals with different histories of exposure to PSP and exposed to toxic and non-toxic diets (3 replicates per experimental group at each sampling time). A, Melinka, Aysén (with previous PSP exposure); B, Corral, Valdivia (without previous PSP exposure).

**Figure 5:**
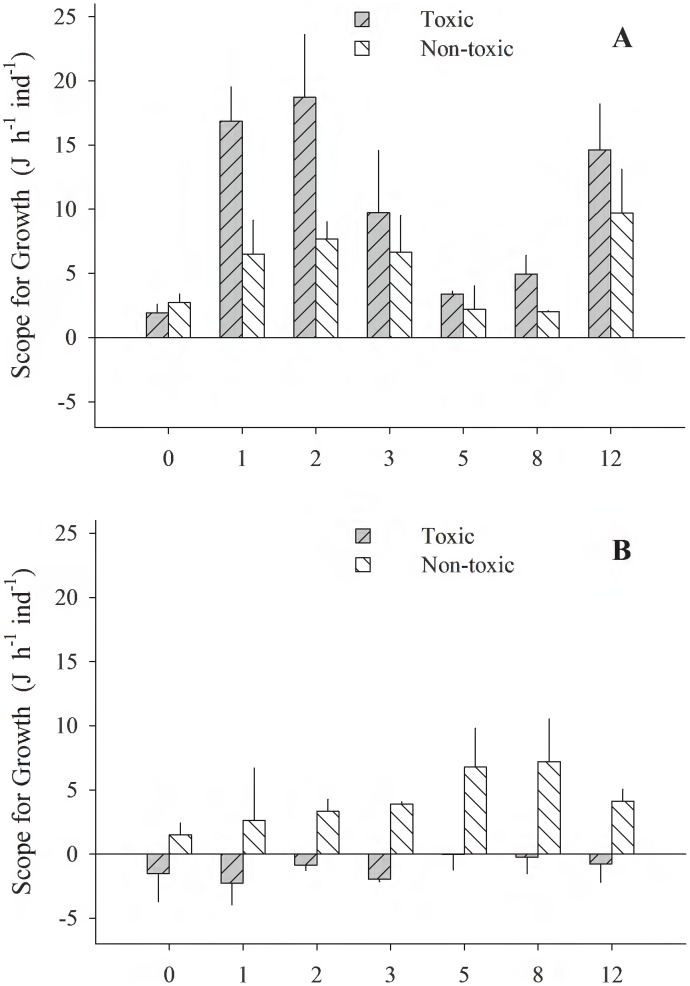
*Tagelus dombeii*. Scope for growth measured for a period of 12 days in individuals with different histories of exposure to PSP and exposed to toxic and non-toxic diets (3 replicates per experimental group at each sampling time). A, Melinka, Aysén (with previous PSP exposure); B, Corral, Valdivia (without previous PSP exposure).

Figures 1–5 (see File S1) illustrate the different physiological processes, CR, AR, VNH_4_-N, VO_2_, and SFG, measured in the 2 populations of *T. dombeii*, in relation to time of exposure (TE) to the toxin and the two diets. Clams from Melinka, Aysén maintain high levels of filtration and absorption during the experimental period, without significant differences (p>0.05) between the toxic and non-toxic groups (Fig 1A, 2A). On the contrary, the clams from Corral, Valdivia exposed to the toxic diet reduced significantly (p<0.05) their clearance and absorption rates (Fig. 1B; 2B). Ammonia excretion did not show significant differences (p>0.05) between the clams exposed to the toxic diet and those fed on the non-toxic diet in both studied populations (Fig. 3 A and B). Oxygen uptake was similar for both groups of clams; however, significant differences were recorded on a few occasions (Fig. 4 A and B). The scope for growth of clams from Melinka, Aysén (10.04±1.72 J h^−1^ ind^−1^), was not affected by diet containing *A. catenella*, accumulating similar or higher amounts of energy than clams from the non-toxic group (Fig. 5 A). Conversely, the scope for growth of the clams of Corral, Valdivia (−1.09±0.47 J h^−1^ ind^−1^) exposed to PSP was negative and significantly lower than in the non-toxic group, during the whole experimental period (the Fig 5 B).

When the three factors, origin, time exposure and diet were included in the analyses, the three-way repeated measure ANOVA (Table 2) showed that the diet did not have a significant (p>0.05) effects on the different physiological processes. By contrast, the origin of the clams was significant (p<0.05) for all physiological variables, and interaction between diet and origin was significant (p<0.05) for CR, AR, and SFG. According to the within-tank analyses, TE and the interaction between TE and the factor origin, showed a significant (P<0.05) effect on all of the physiological variables measured. The interaction between the TE and diet showed a significant (p<0.05) effect only for VO_2_, and the three-way interaction was not significant for all physiological processes measured. The clearance rate, absorption rate and ammonia excretion rate measured in the clams exposed to the toxic diet were significantly affected by the origin of the clams (two-way ANOVA repeated-measured, p<0.05; Table 3), with significantly lower values for the individuals from Corral-Valdivia (Table 4). The physiological index scope for growth for the specimens from Corral-Valdivia was also significantly affected (p<0.05) by the diet containing PSP, resulting in negative values (−1.09±0.47 J h^−1^ ind^−1^) compared to the high values (10.04±1.72 J h^−1^ ind^−1^) for the specimens from Melinka-Aysén (Table 3).

**Table 2:** Three-way repeated-measures ANOVA for clearance rate, absorption rate, ammonia excretion, oxygen uptake and scope for growth in the razor clam *Tagelus dombeii*. TE = Time exposure.

Error: Tank	Diet			Origin			Diet:Origin					
	*df*	F	p	*df*	F	p	*df*	F	p			
Clearance Rate	1	0.483	0.506	1	33.476	0.00041 ***	1	9.314	0.016 *			
Absorption Rate	1	0.039	0.848	1	37.296	0.00028 ***	1	13.175	0.0067 **			
Ammonia Excretion	1	0.026	0.875	1	57.970	6.22·10^−05^ ***	1	2.235	0.173			
Oxygen Uptake	1	0.304	0.596	1	11.708	0.00906 **	1	3.175	0.112			
Scope for Growth	1	0.074	0.792	1	28.813	0.000671 ***	1	19.026	0.0024 **			

Error: Within	TE			TE:Diet			TE:Origin			TE:Diet:Origin		
	*df*	F	p	*df*	F	p	*df*	F	p	*df*	F	p
Clearance Rate	6	7.339	1.33·10^−05^ ***	6	1.203	0.321	6	7.014	2.13·10^−05^ ***	6	1.009	0.430
Absorption Rate	6	5.952	0.0001 ***	6	1.073	0.392	6	8.442	2.87·10^−06^ ***	6	0.649	0.690
Ammonia Excretion	6	6.474	4.74·10^−05^ ***	6	0.802	0.574	6	9.981	3.86·10^−07^ ***	6	0.801	0.574
Oxygen Uptake	6	11.980	3.55·10^−08^ ***	6	3.469	0.0063 **	6	3.401	0.0071 **	6	1.144	0.352
Scope for Growth	6	4.011	0.00247 **	6	1.267	0.290	6	6.150	7.75·10^−05^ ***	6	1.036	0.414

* p<0.05.

** p<0.01.

*** p<0.001.

**Table 3:** Two-way repeated-measures ANOVA for clearance rate, ammonia excretion, oxygen uptake and scope for growth in the razor clam *Tagelus dombeii*. TE = time of exposure.

Physiological Rate	Origin				TE				TE:Origin			
	*df*	F	p		*df*	F	p		*df*	F	p	
Clearance Rate	1	21.387	0.0098	**	6	4.620	0.0029	**	6	6.157	0.0005	***
Ammonia Excretion	1	40.184	0.0031	**	6	4.812	0.0023	**	6	8.933	0.0000	***
Oxygen Uptake	1	0.878	0.401		6	14.409	0.0000	***	6	2.981	0.0254	**
Scope for Growth	1	31.676	0.0049	**	6	4.747	0.0025	**	6	5.651	0.0009	***

* *p*<0.05.

** *p*<0.01.

*** *p*<0.001.

**Table 4:** Physiological variables (mean ± standard error) of specimens of *Tagelus dombeii* from two populations with different history of exposure to PSP.

	Corral-Valdivia		Melinka-Aysén	
Physiological Rate	Toxic	Non-toxic	Toxic	Non-toxic
Clearance Rate (L h^−1^ ind^−1^)	0.31±0.02	0.54±0.06	0.87±0.09	0.72±0.07
Absorption Rate (mg h^−1^ ind^−1^)	0.23±0.02	0.46±0.03	0.80±0.09	0.60±0.06
Ammonia Excretion (µg NH_4_-N h^−1^ ind^−1^)	17.82±2.83	23.43±3.16	56. 13±4.89	49.16±4.46
Oxygen Uptake (mL h^−1^ ind^−1^)	0.28±0.02	0.24±0.02	0.30±0.02	0.32±0.03
Scope for Growth (J h^−1^ ind^−1^)	−1.09±0.47	4.21±0.87	10.04±1.72	5.36±0.93

**Figure 6:**
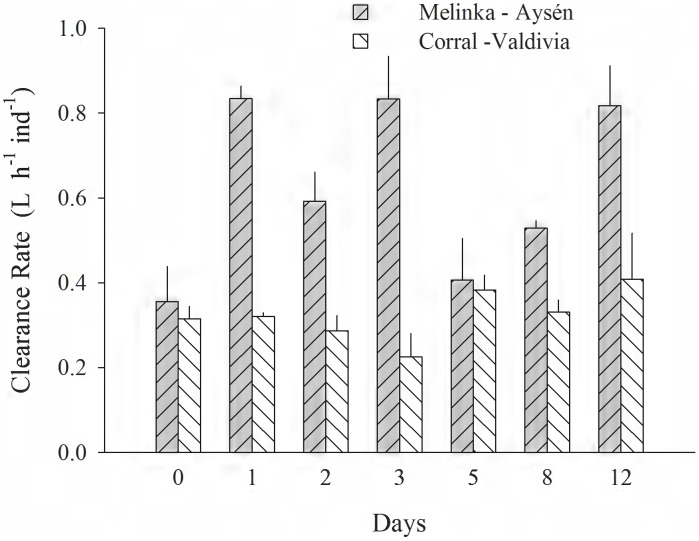
*Tagelus dombeii*. Clearance rate measured on *Alexandrium catenella* cells for a period of 12 days in individuals with different histories of exposure to PSP. A, Melinka, Aysén (with previous PSP exposure); B, Corral, Valdivia (without previous PSP exposure).

Figure 6 (see File S1) shows the comparison of the *T. dombeii* clearance rate for *A. catenella* only. The one-way ANOVA showed that the clams from Corral-Valdivia had significantly (P<0.05) lower clearance rates (0.33±0.05 L h^−1^ ind^−1^) than the individuals from Melinka-Aysén (0.62±0.07 L h^−1^ ind^−1^).

## Discussion

The *Tagelus dombeii* clams from Melinka-Aysén, which are frequently exposed to PSP, were not affected by the presence of toxin in the diet, unlike the population from Corral-Valdivia, which is not exposed to PSP. The clams from Corral-Valdivia showed significantly reduced filtration activity and absorption, which affected the amount of energy channeled to growth and reproduction (SFG). Previous studies have shown similar results for other species of filter-feeder organisms [7], [31], [32], [33], [34].

The effect of PSP on bivalve filter feeders can vary intra and inter specifically, depending on multiple factors, such as toxicity of the algae, differences in digestive functions and the history of exposure to toxic algae blooms [35], [9], [36]. *Crassostrea gigas* presents a complete inhibition of filtration activity during the first hours of exposure to a diet containing *Alexandrium tamarense* [37]. However, this species requires two weeks to initiate normal feeding activity on a diet containing the dinoflagellate *A. catenella* [38]. The greater filtering activity of specimens from Melinka-Aysén during the intoxication phase (fig. 1 A) is explained by their history of frequent exposure to natural blooms of *A. catenella*. Therefore, the capacity of *T. dombeii* from Melinka-Aysén to ingest *A. catenella*, coupled with increased enzymatic activity to degrade the toxic cells as described in a parallel study by Fernández-Reiriz et al. [15], suggest an adaptation by which this population can use the toxic *A. catenella* as a food resource. Studies of the clam *Mya arenaria* [9], [39] and the mussel *M. edulis* [40], [41] with different histories of PSP exposure followed by exposure to *A. tamarense* are consistent with the results of the present study. This adaptation would respond to structural changes at the molecular level, in which resistance is attributed to natural mutations in the sodium channels of the bivalves after exposure to frequent PSP events [9]. The lower absorption rates of clams from Corral-Valdivia fed the toxic diet (Fig. 2 B) may be related to impaired digestive processes, similar to those described by Wikfors and Smolowitz [42] and Smolowitz and Shumway [43] in the scallop *Argopecten irradians* fed the toxic dinoflagellates *Gyrodinium aureolum* and *Prorocentrum minimum*. According to these authors, these dinoflagellates produce cytotoxicity and necrosis of the cells responsible for the absorption of food. Widdows et al. [44] also described cellular damage in the digestive tract of the mussel *Mytilus edulis* fed *Gyrodinium aureolum*. These results are consistent with the steady decline in the absorption rate of *T. dombeii* from Corral-Valdivia during the intoxication period, with negative consequences from an energetic standpoint. The higher ammonium excretion rate of toxic specimens from Melinka-Aysén may be related to their greater capacity to degrade the paralyzing toxin, which is a rich source of nitrogen [45]. Navarro and Contreras [6] described a similar response for the mussel *Mytilus chilensis* from a population with history of exposure to PSP (Yaldad Bay, Chiloé). Degradation of the toxin produces high concentrations of nitrogen products, which must be removed from the body to maintain the osmotic balance of the bivalve. Therefore, *T. dombeii* controls the excess nitrogen contained in the toxic diet, thereby maintaining physiological stability against high concentrations of toxic dinoflagellates.

The toxic diet did not affect the oxygen consumption of the two populations of *T. dombeii*. Similar responses were obtained with *M. chilensis* from southern Chile when exposed to a diet containing *A. catenella* [6]. However, various responses have been described for other species of bivalves. The scallop *Placopecten magellanicus* and the clam *Spisula solidissima* showed a decrease in oxygen consumption, in contrast to the increase shown by the bivalves *Mya arenaria* and *Mytilus edulis* [4]. According to Marsden and Shumway [46], the mussel *Perna canaliculus* exposed to *A. tamarense* also showed significantly increased oxygen consumption. This makes evident the existence of a species-specific effect of paralyzing toxin on oxygen consumption.

Several studies have reported a negative effect of toxic algal blooms on the growth rate of various species of filter feeder bivalves [5], [6]. Widdows [47] and Navarro and Winter [48] obtained values for the scope for growth of 15 and 10 J h^−1^ g^−1^ for individuals of *M. edulis* and *M. chilensis*, respectively, which were fed monocultures of non-toxic microalgae. The present study shows similar values for individuals of similar sizes from Melinka-Aysén for both the non-toxic group and the group exposed to PSP (ca.10 J h^−1^ ind^−1^). However, the scope for growth of *T. dombeii* from Corral-Valdivia was negative (−1.09±0.47 J h^−1^ ind^−1^) when the specimens were exposed to the toxic diet, similar to that described by Li et al. [5] for the clam *Ruditapes philipinarum* (−6.2±2.8 J h^−1^ g^−1^). The non-toxic groups of both populations showed no significant differences between the values of SFG, suggesting that the differences between the two populations exposed to PSP are due to different responses to *A. catenella* associated with the history of exposure to the dinoflagellate. Our results are consistent with those of MacQuarrie [49] and Bricelj et al. [9], who described the different behavioral and physiological response of the clam *M. arenaria* to *A. tamarense*, depending on their prior history of exposure to PSP. Therefore, *T. dombeii* specimens from populations with no history of exposure to PSP show a greater sensitivity to the presence of STX in the diet by reducing their feeding and growth rates compared to individuals from populations that experience frequent exposure to PSP events. Thus, the presence of PSP in the natural environment may have a potential negative effect on the broodstock of the clam from Corral-Valdivia. In *Mytilus edulis* [50] and *Ostrea chilensis* [51] it has been observed that stress feeding conditions reduce fecundity and quality of the eggs, with a smaller number of larvae being obtained.

According to the present study, clams from the Melinka-Aysén population apparently do not suffer negative consequences from the toxin produced by *A. catenella*; an adaptive response to the frequent blooms of this dinoflagellate that occur in their environment. This contrast with that observed for *T. dombeii* specimens with no history of exposure to *A. catenella*, which were affected by exposure to diets containing PSP, with a large reduction in the energy allocated to growth. The present study suggests that the history of exposure to PSP plays an important role in the physiological performance and fitness of filter feeding bivalves.

## Supporting Information

File S1 **Data for the different physiological variables measured are included in the file S1.**
(XLSX)
